# A Neural Circuit From Paraventricular Nucleus of the Thalamus to the Nucleus Accumbens Mediates Inflammatory Pain in Mice

**DOI:** 10.1002/brb3.70218

**Published:** 2024-12-31

**Authors:** Xi Liu, Xi Zhang, Dongxu Wang, Ya Cao, Ling Zhang, Zhonghua Li, Qin Zhang, Yu Shen, Xian Lu, Keyu Fan, Mingxia Liu, Jingqiu Wei, Siping Hu, He Liu

**Affiliations:** ^1^ Department of Anesthesiology & Clinical Research Center for Anesthesia and Perioperative Medicine & Key Laboratory of Anesthesia and Analgesia Application Technology Huzhou Central Hospital, The Fifth School of Clinical Medicine of Zhejiang Chinese Medical University Huzhou China; ^2^ Department of Anesthesiology & Clinical Research Center for Anesthesia and Perioperative Medicine & Key Laboratory of Anesthesia and Analgesia Application Technology Huzhou Central Hospital, The Affiliated Central Hospital of Huzhou University Huzhou China; ^3^ Department of Anesthesiology & Clinical Research Center for Anesthesia and Perioperative Medicine & Key Laboratory of Anesthesia and Analgesia Application Technology Affiliated Huzhou Hospital, Zhejiang University School of Medicine Huzhou China; ^4^ Department of Anesthesiology Hangzhou Hospital of Traditional Chinese Medicine Hangzhou China; ^5^ School of Brain Science and Brain Medicine Zhejiang University School of Medicine Hangzhou China; ^6^ Department of Education & Training, Huzhou Central Hospital The Fifth School of Clinical Medicine of Zhejiang Chinese Medical University Hangzhou China

**Keywords:** inflammatory pain, nucleus accumbens, optogenetics, paraventricular nucleus of the thalamus

## Abstract

**Background:**

Pain is a prevalent comorbidity in numerous clinical conditions and causes suffering; however, the mechanism of pain is intricate, and the neural circuitry underlying pain in the brain remains incompletely elucidated. More research into the perception and modulation of pain within the central nervous system is essential. The nucleus accumbens (NAc) plays a pivotal role in the regulation of animal behavior, and extensive research has unequivocally demonstrated its significant involvement in the occurrence and development of pain. NAc receives projections from various other neural nuclei within the brain, including the paraventricular nucleus of the thalamus (PVT). In this experiment, we demonstrate that the specific glutamatergic neural circuit projection from PVT to NAc (PVT^Glut^→NAc) is implicated in the modulation of inflammatory pain in mice.

**Methods:**

We compared the difference in pain thresholds between complete Freund's adjuvant (CFA)‐induced inflammatory pain models and controls. Then in a well‐established mouse model of CFA‐induced inflammatory pain, immunofluorescence staining was utilized to evaluate changes in c‐Fos protein expression within PVT neurons. To investigate the role of PVT^Glut^→NAc in the modulation of pain, we used optogenetics to modulate this neural circuit, and nociceptive behavioral tests were employed to investigate the functional role of the PVT^Glut^→NAc circuit in the modulation of inflammatory pain.

**Results:**

In the mice with the inflammatory pain group, both the paw withdrawal latencies (PWLs) and paw withdrawal thresholds (PWTs) of the right hind paw were decreased compared to the control group. In addition, compared to the control group, CFA‐induced inflammatory pain led to increased c‐Fos protein expression in PVT, which means that some of the neurons in this area of the brain region have been activated. Following the injection of retrograde transport fluorescent‐labeled virus into NAc, glutamatergic neurons projecting from the PVT to NAc were observed, confirming the projection relationship between PVT and NAc. In the experiments in optogenetic regulation, normal mice exhibited pain behavior when the PVT^Glut^→NAc circuit was stimulated by a 473 nm blue laser, resulting in decreased PWLs and PWTs compared to the control group, which means activating this neural circuit can lead to painful behaviors. In the CFA‐induced pain group, inhibition of the PVT^Glut^→NAc circuit by a 589 nm yellow laser alleviated pain behavior, leading to increased PWLs and PWTs compared to the control group, representing the fact that inhibition of this neural circuit relieves pain behaviors.

**Conclusions:**

The findings unveil a pivotal role of the PVT^Glut^→NAc circuit in modulating inflammatory pain induced by CFA in mice.

## Graphical abstract

Schematic summary of the hyperactivity of the PVT^Glut^→NAc circuit in modulating inflammatory pain. In the state of inflammatory pain induced by complete Freund's adjuvant, the hyperactivity of the PVT→NAc projection circuit plays a role in regulating inflammatory pain, with the specific involvement of glutamatergic neurons in the PVT projecting to the NAc as the cellular mechanism mediating the regulatory function.

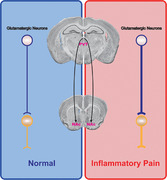

**Abbreviation**
BL: baseline
CCI: chronic constriction injury
ChR2: channelrhodopsin‐2
Cre: cyclization recombination enzyme
DIO: double‐floxed inverse orientation
Glu: glutamate
IASP: International Association for the Study of Pain
mPFC: medial prefrontal cortex
NAc: nucleus accumbens
NpHR: halorhodopsin
PBS: phosphate‐buffered saline
PFC: prefrontal cortex
PVT: paraventricular thalamic nucleus
PWLs: paw withdrawal latencies
PWTs: paw withdrawal thresholds
TBS: tris buffered saline
vHIP: ventral hippocampus
VTA: ventral tegmental area

## 1 Introduction

The management of pain poses a substantial economic burden and hampers functionality within modern healthcare, manifesting prominently across various chronic and acute medical conditions (Cohen, Vase, and Hooten 2021). The sensation of pain serves as a symptom of various acute and chronic health conditions while also being recognized as an independent ailment. According to the updated definition by the International Association for the Study of Pain (IASP) in 2020, pain is characterized as an unpleasant sensory and emotional experience associated with, or resembling that associated with, actual or potential tissue damage (Raja et al. 2020), which marks the first revision of the IASP's globally utilized definition since 1979. The experience of pain can have adverse impacts on physical functionality, mental well‐being, social relationships, and overall social health (Sturgeon and Zautra 2016). The advancements in brain science technologies have led an increasing number of researchers to propose that the perception and regulation of pain is a physiological response generated by intricate neural networks within the nuclei of the brain, following sophisticated information processing, in response to noxious stimuli (Yam et al. 2018). Nevertheless, the intricacies of pain mechanisms persist while our understanding of the neural circuitry involved in brain‐based pain processing remains incomplete. Therefore, it is crucial to undertake additional investigations into perceiving and regulating pain within the central nervous system.

The nucleus accumbens (NAc) is one of the crucial brain regions involved in the regulation of animal behaviors. The existing literature suggests that the NAc is a complex structure that primarily participates in cognitive processes, drug addiction, aversion responses such as disgust and fear, as well as reward‐related behaviors (Neumann et al. 2016; Zhu et al. 2016). The advancement of research has led to a growing body of evidence substantiating the pivotal role played by NAc in the initiation and progression of pain (Baliki et al. 2010; P.‐C. Chang et al. 2014; Descalzi et al. 2017; Goffer et al. 2013; Kai et al. 2018; Kc et al. 2020; Lee et al. 2015; Makary et al. 2020; Su et al. 2016; Wu et al. 2018; Xu et al. 2015; H. Zhang et al. 2017; Y. Zhang et al. 2019; Zhou et al. 2018). The NAc projects to and exchanges information with relevant pain‐related structures, including the prefrontal cortex (PFC), anterior cingulate cortex, periaqueductal gray, habenular nucleus, thalamus, and so forth (Harris and Peng 2020). In addition, the ventral tegmental area (VTA) is a crucial brain region involved in the modulation of pain and depression (Bannister et al. 2017; Kato et al. 2016; Porreca and Navratilova 2017). The inhibition of mesolimbic dopaminergic neuron activity is likely to contribute to a reduction in the inhibition of output neurons in the NAc, potentially playing a role in neuropathic or cancer pain (Watanabe et al. 2018). The dopaminergic neurons projecting from the VTA to the NAc exhibit an elevation in spontaneous electrical activity during chronic neuropathic pain induced by chronic compressive injury (CCI). Furthermore, optogenetic inhibition of VTA→NAc projective neuronal firing demonstrates a potential alleviation of thermal hyperalgesia in the animal models of chronic neuropathic pain induced by CCI (H. Zhang et al. 2017). The role of the VTA→NAc circuit in regulating chronic pain and depression has been further validated by another study, providing compelling evidence for the involvement of VTA→NAc dopaminergic projection in mediating the pain process (D. Liu et al. 2018).

In addition to receiving dopaminergic input from VTA, NAc also receives glutaminergic input from other nuclei, such as the paraventricular nucleus of the thalamus (PVT), PFC, and ventral hippocampus (Neumann et al. 2016; Zhu et al. 2016; Bagot et al. 2015; Britt et al. 2012; Christoffel et al. 2015). The PVT is a midline structure primarily composed of glutamatergic neurons, functioning as the body's sentinel to detect and respond to both external physical and mental stimuli (Matzeu et al. 2018; Penzo et al. 2015), and it plays a pivotal role in stress regulation, vigilance maintenance, wakefulness promotion, memory formation and retrieval, cognitive processes facilitation, motivational behavior modulation, as well as the integration of sensory and pain information (Millan, Ong, and Mcnally 2017). It also illuminated the roles of PFC and PVT in the affective and mechanical components of visceral nociception (Jurik et al. 2015). The application of foot pinching enhances the expression of c‐Fos protein in the PVT, suggesting potential involvement of PVT in the pain processing pathway (Ehling et al. 2018). The involvement of PVT in pain regulation remains ambiguous despite recent studies highlighting its potential role in chronic neuropathic pain (Cheng et al. 2017).

The glutamatergic inputs received by the NAc from PVT, and both brain regions have been implicated in mediating information processing of pain sensation independently, but the functional contribution of the PVT^Glut^→NAc circuit has never been directly investigated in the modulation of inflammatory pain. Our preliminary experimental findings suggest that plantar injection of complete Freund's adjuvant (CFA) can induce an upregulation in the expression of c‐Fos in PVT.

In the current study, we demonstrate that a glutamatergic circuit from PVT to NAc (PVT^Glut^→NAc) undergoes maladaptive changes by using a well‐established mouse model of a CFA‐induced inflammatory pain. Furthermore, we demonstrate that optogenetic activation of the PVT^Glut^→NAc circuit induces nociceptive behaviors in sham mice, and optogenetic inhibition of the PVT^Glut^→NAc circuit alleviates nociceptive behaviors in the CFA‐induced inflammatory pain model. Together, our results reveal the functional role of the PVT^Glut^→NAc circuit in the modulation of inflammatory pain.

## 2 Materials and Methods

### 2.1 Animals

Male C57BL/6J mice (procured from Hangzhou Hangsi Biotechnology Co. Ltd), aged 8–12 weeks, were used in all experiments of this study. All mice were housed in cages equipped with a dedicated ventilation system under standard laboratory conditions (12 h light/12 h dark cycle, lights on 08:00 a.m. to 08:00 p.m., temperature of 23°± 2°C, and humidity of 50% ± 10%) with ad libitum access to standard lab mouse pellet food and water. All animal care, use, and procedures in this study were approved by the Ethical Review Committee of Laboratory Animal Welfare of Huzhou Central Hospital and conformed to the ethical guidelines for animal experimentation, and all experimental protocols were conducted according to the National Institute of Health Guide for Care and Use of Laboratory Animals (IACUC Protocol: 150005A2). The experimental protocols and the functional tests employed in this study were meticulously designed to minimize the utilization of animals and mitigate any potential discomfort they may experience. We eliminated the mice that did not get the virus correctly and ended up with a total of 53 mice in our study.

### 2.2 Inflammatory Pain Model

A volume of 25 µL of CFA was subcutaneously injected into the right hind paw with a 20‐gauge micro‐injector to establish a mouse model of inflammatory pain (Nagakura et al. 2003).

### 2.3 Pain Behavioral Tests

We assessed the pain severity in mice by measuring their thermal and mechanical pain thresholds. Investigators responsible for the behavioral test were blinded to which animals represented treatments or controls.

The Hargreaves method was employed to determine the thermal pain threshold (Hargreaves et al. 1988). The mice were individually habituated in transparent acrylic enclosures on an elevated glass table in a temperature‐controlled and noise‐free room, allowing for 1 h of habituation before the test. A mobile heat‐producing radiant heat source was focused on their right hind paw to stimulate the plantar surface. The paw withdrawal latencies (PWLs) were defined as the time from the light‐on to a paw withdrawal or paw licking being recorded. The basic PWLs of mice predominantly remained 15–20 s by adjusting the radiant light intensity, and this intensity remained constant throughout the duration of the experiment. The stimulus instrument was programmed with an automatic cut‐off time of 20 s to prevent thermal radiation‐induced tissue damage. The PWLs were measured for 5 repeats/time points/animal, removing the maximum and minimum values and reserving the last three for analysis.

The mechanical pain threshold of the mice was evaluated using the von Frey test as previously described (Chaplan et al. 1994). The mice were acclimated in transparent acrylic enclosures on a wire mesh platform in a temperature‐controlled, quiet room for 1 h before the commencement of the experiment. Similarly, during a period of relative quiescence and reduced engagement in exploratory and other activities, each von Frey filament (ranging from 0.008, 0.02, 0.04, 0.07, 0.16, 0.4, 0.6, 1.0, and 1.4 g with logarithmically incremental stiffness) was applied perpendicularly to the plantar surface of the right hind paw with sufficient force to bend the filament. The von Frey filaments were kept for a duration of 4–6 s or until eliciting a paw withdrawal response to assess the paw withdrawal thresholds (PWTs). Lifting, shaking, or licking the paw indicated a positive response and prompted the next weaker filament, and the absence of a paw withdrawal response prompted the next stronger filament. Three positive behaviors were recorded as the mice's mechanical pain threshold. The von Frey filament, which made mice three positive behaviors, was recorded as the mice's PWTs.

### 2.4 Surgical Procedures

Brain stereotaxic surgeries and injections were conducted following the previously established protocols (D.‐B. Li et al. 2019; X. Li et al. 2021; H. Liu et al. 2021). The mice were immobilized in a stereotaxic apparatus (RWD Life Technology Co. Ltd., Shenzhen, China) under general anesthesia with 1% pentobarbital sodium (40 mg/kg, dissolved in saline, ip). All the viruses were purchased from Brain VTA Technology Co. Ltd. (Wuhan, China). The corresponding viruses were injected into the PVT (unilateral coordinates, Bregma: AP: −1.4 mm; LM: +0.1 mm; DV: −3.0 mm, 4°) and the NAc (bilateral coordinates, Bregma: AP: +1.4 mm; LM: ±0.8 mm; DV: −4.6 mm, 0°) using glass pipettes with a tip diameter of approximately 20 µm controlled by a programmable nanoliter injector. The stereotaxic coordinates mentioned in this study are based on the stereotaxic atlas of The Mouse Brain in Stereotaxic Coordinates (the second edition, George Paxinos and Keith B.J. Frank edited). The speed of virus injection was 100 nL/min and the pipette tip was left in place at the injection site for 10 min post‐injection before being slowly withdrawn. To illuminate the PVT^Glut^→NAc pathway, the optical fiber implantations were performed using a stereotaxic apparatus. The optical fibers (ceramic ferrule: diameter 2.50 mm; optical fiber: 200 µm core diameter, 0.39 NA, FT200EMT, Thorlabs) were precisely implanted in the PVT (unilateral coordinates, Bregma: AP: −1.4 mm, LM: −0.1 mm, DV: −3.0 mm, 4°). The optical fibers were securely affixed to the skull using dental cement. All mice were given a recovery period of 3–4 weeks after the surgery, during which all viruses were adequately expressed.

### 2.5 Optogenetics

The technique of optogenetics enables the targeted investigation of neuronal circuitry and precise manipulation of behavioral responses (Xie, Wang, and Bonin 2018). In the optogenetic experiments, retrograde recombination adeno‐associated cyclization recombination enzyme (Cre) virus‐encoded the broad‐spectrum promoter CaMKIIα (rAAV‐CaMKIIα‐Cre) into the NAc. This allowed rAAV encoding Cre recombinase‐inducible channelrhodopsin (rAAV‐EF1α‐DIO‐ChR2‐EYFP) or rAAV encoding Cre recombinase‐inducible enhanced halorhodopsin 3.0 (rAAV‐EF1α‐DIO‐NpHR3.0‐EYFP) or rAAV encoding Cre‐dependent control EYFP virus (rAAV‐EF1α‐DIO‐EYFP) injected into the PVT to express, targeting PVT^Glut^→NAc projecting neurons and their axon terminals. When the viruses were adequately expressed, a fiber core implanted in the mouse brain was connected to a combined laser generator and the stimulator (Newdoon, Hangzhou, China), used to generate a 473 nm wavelength of the blue laser or 589 nm of the yellow laser. In the behavioral experiments, the parameters for blue light stimulation were as follows: wavelength of 473 nm, frequency of 20 Hz, wave width of 10 ms, stimulation energy of 10 mW, and a single stimulation lasting for 1 h for activating PVT neurons in normal mice; the PVT neurons in CFA inflammatory pain mice were inhibited using yellow light and the stimulation parameters were as follows: wavelength of 589 nm, frequency of 0.1 Hz, 8‐s‐on/2‐s‐off, stimulation energy of 10 mW, and a single stimulation lasting for 2 h.

### 2.6 Brain Tissue Preparation

The mice were deeply anesthetized using pentobarbital sodium (40 mg/kg) and then perfused transcardially with phosphate‐buffered saline (PBS) until a yellowing of the liver was observed and sequentially perfused with 4% paraformaldehyde. The brains were removed, collected, and postfixed in 4% paraformaldehyde for 6–8 h, followed by dehydrated in 20% sucrose at 4°C overnight. Subsequently, they were transferred to a solution of 30% sucrose at 4°C overnight again. The brains of mice were preserved by freezing them in optimal cutting temperature compound Tissue‐Tek OCT. Subsequently, the frozen brains were sliced coronally into sections at a 30 µm thickness by using a cryotome (VT1000S, Leica Microsystems). These sections were then collected in PBS for staining and observation purposes.

### 2.7 Immunofluorescence

The use of c‐Fos as a neuronal activation marker is widespread, and its strengths lie in low expression levels under basal conditions and rapid induction within minutes after acute challenges or stimuli, reaching maximal levels (Kovács 1998). The immunofluorescence staining was performed as follows. First, the floating brain sections were washed thrice with PBS and twice with 0.4% Tris‐buffered saline (TBS) followed by blocking for 1 h in 1% donkey serum diluted in 0.4% TBS at room temperature. Subsequently, the sections were incubated overnight at 4°C with primary rabbit anti‐c‐Fos polyclonal antibody (c‐Fos (9F6) Rabbit mAb, 1:1000, Cell Signaling Technology, USA). The following day, the sections were washed three times in TBS after being rewarmed for 2 h at room temperature. Subsequently, they were incubated with secondary biotinylated goat anti‐rabbit antibody (IgGH&L Alexa Fluor594, 1:1000; Abcam, UK) for 2 h at room temperature and then overnight at 4°C. After being washed three times with PBS, the brain section slices were mounted on the slide glass. The slides were air‐dried and sealed using anti‐fluorescence quenching DAPI sealing tablets before being stored in a dark environment at −20°C. The visualization and quantification of c‐Fos positive neurons were conducted utilizing fluorescence microscopy.

### 2.8 Statistical Analysis

The estimated sample sizes were based on our past experience performing similar experiments. GraphPad Prism version 8.0 software was utilized for the statistical analysis of experimental data and the generation of statistical charts. The results were presented as means ± standard errors of the means (SEM). For the assessment of acute inflammatory pain mice and normal mice, a two‐way ANOVA was employed to compare the PWLs and PWTs among different groups. Unpaired two‐tailed *t*‐test was used for comparing two groups with normally distributed data. Statistical significance was considered at *p* values below 5% probability.

## 3 Results

### 3.1 Alterations in Nociceptive Behaviors of Mice With Inflammatory Pain

**FIGURE 1:**
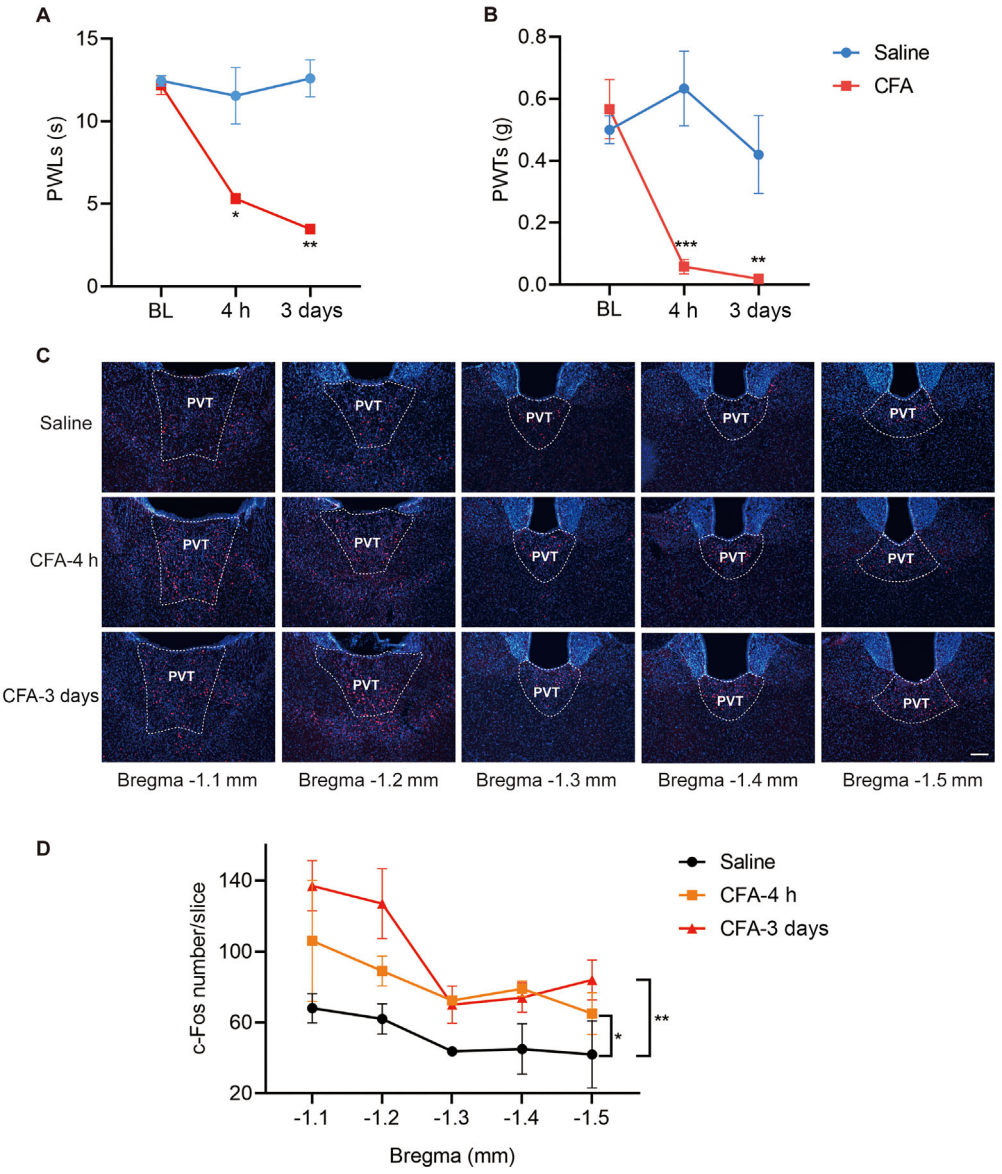
Changes of pain behaviors and c‐Fos at 4 h and 3 days in inflammatory pain mice. (A, B) PWLs and PWTs of saline and CFA mice (mean ± SEM; two‐way ANOVA; *p < 0.05, **p < 0.01, ***p < 0.001, *n* = 6, six mice). The PWLs and PWTs in mice injected with CFA were significantly reduced. (C) Coronal sections at different levels showed the expression of c‐Fos protein in PVT brain regions of mice in the saline group, CFA 4 h group, and CFA 3‐day group (scale bar = 100 µm). (D) Quantitative analysis of c‐Fos protein expression in PVT brain regions (mean ± SEM; *t*‐test *p < 0.05, **p < 0.01).

We first observed the alterations in nociceptive behaviors of mice with inflammatory pain induced by intra‐plantar injection of CFA. The experimental group was administered CFA to induce inflammatory pain, and the control group was injected with saline, followed by separate assessments of thermal and mechanical pain sensitivity in normal mice and those with inflammatory CFA injection at 4 h and 3 days post‐injection. Statistical analysis revealed that compared to the control group, the PWLs and PWTs of the right hind paw in mice injected with CFA were significantly reduced (*n* = 6, 6) (Figure 1A,B).

### 3.2 The Expression of c‐Fos in the PVT Is Upregulated in the Inflammatory Pain Mice Model

To investigate the activation of neurons in the PVT under conditions of inflammatory pain, CFA was administered to the experimental group via injection into the right hind paw of the mice, while saline was injected into the right hind paw of the mice in the control group, and the brain tissue samples were collected from the mice after they were sacrificed after 4 h and 3 days post‐injection, separately. The expression of c‐Fos in PVT was detected by immunoluminescence. The results revealed there was an increase in c‐Fos protein expression within this area among mice experiencing persistent inflammatory pain induced by CFA (Figure 1C,D), which means that some of the neurons in this area of the brain region have been activated.

### 3.3 The PVT Comprises Glutamatergic Neurons That Project to the NAc

**FIGURE 2:**
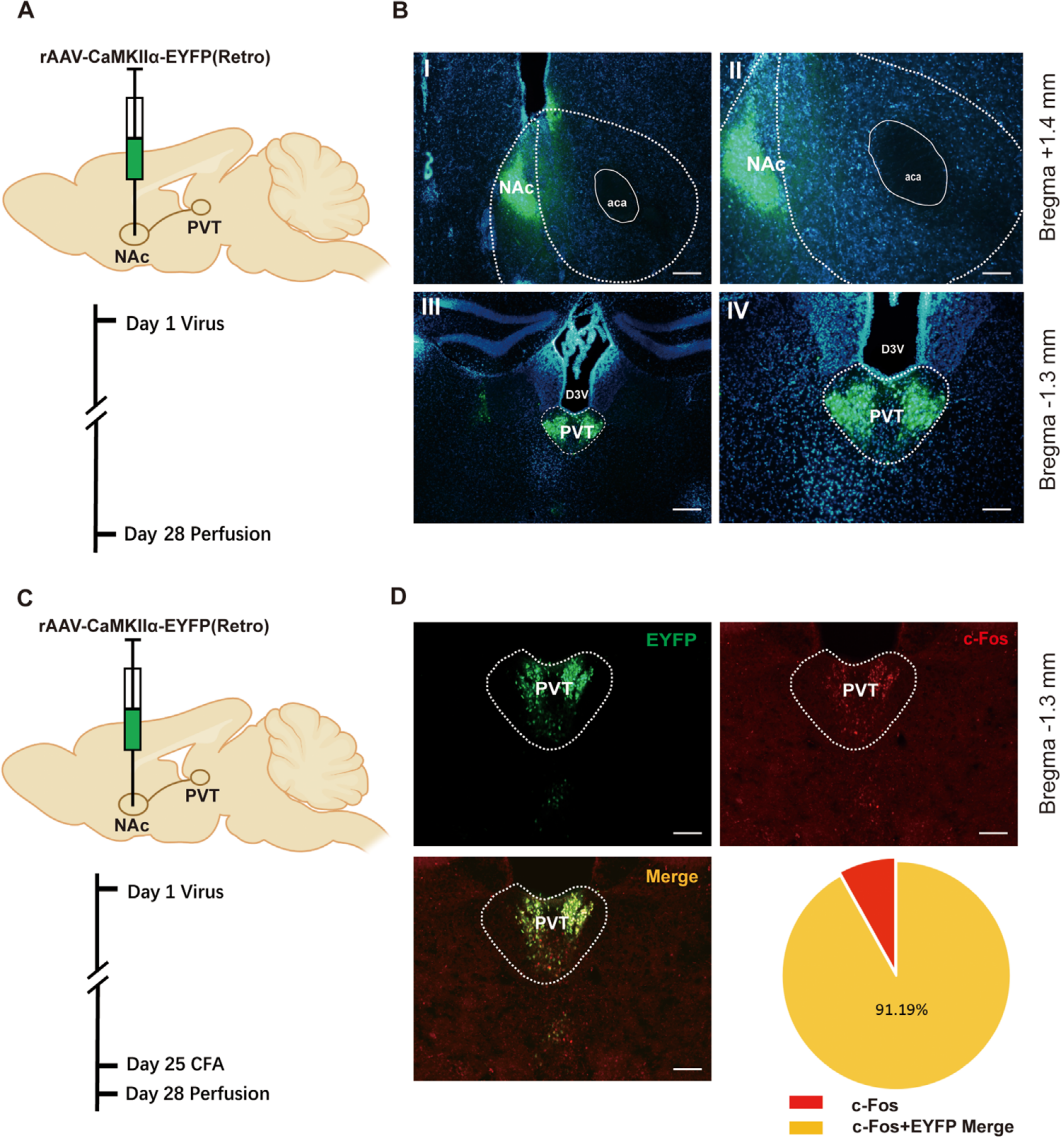
The reverse transport fluorescently labeled virus injecting in the NAc brain region was expressed in PVT glutamatergic cell bodies, and most of them were c‐Fos protein positive expression. (A) Schematic diagram of virus injection and the experimental procedure schedule. (B) Reverse transport of fluorescently labeled viruses injecting in NAc (AP: +1.4 mm; LM: ±0.8 mm; DV: −4.6 mm, 0°) expressed in the NAc axon terminals and PVT (Bregma: −1.3 mm) glutamatergic cell bodies (I and III, scale bar = 200 µm; II and IV, scale bar = 100 µm), demonstrating the glutamatergic neurons in the PVT projecting to the NAc. (C) Schematic diagram of virus injection and the experimental procedure schedule. (D) The c‐Fos protein expression in the PVT brain region with fluorescently labeled virus retrograde projection of NAc to PVT glutamatergic neurons (scale bar = 100 µm), indicating a potential association between the PVT^Glut^→NAc circuit and inflammatory pain.

To prove the anatomic projection relationship between the PVT and the NAc, we specifically labeled the PVT glutamatergic neurons projected to the NAc by injecting the retrograde tracer fluorescently labeled virus rAAV‐CaMKIIa‐EYFP (Figure 2A). After 28 days of virus injection, brain tissues were collected from the sacrificed mice. Images showed that the reverse transport fluorescently labeled virus injected in the NAc was expressed in PVT glutamatergic cell bodies. The virus was detected in the cell bodies of glutamatergic neurons in the PVT and their axon terminals in the NAc, providing further structural evidence for a substantial population of glutamatergic neurons in the PVT projecting to the NAc (AP: +1.4 mm; LM: ±0.8 mm; DV: −4.6 mm, 0°) (Figure 2B). Subsequently, we specifically labeled the PVT glutamatergic NAc by injecting the retrograde tracer fluorescently labeled virus rAAV‐CaMKIIa‐EYFP using the same methodology (Figure 2C). After 25 days post‐virus injection, CFA was administered into the right hind paw of the mice. Three additional days later, brain tissues were collected from the sacrificed mice for subsequent analysis of c‐Fos expression in the PVT using immunoluminescence. The c‐Fos immunoreactive cells were visualized in red, while the glutamatergic neurons projecting to the NAc within the PVT were labeled in green. Finally, we discovered that approximately 91.19% of the neurons were co‐labeled, indicating a potential association between the PVT^Glut^→NAc circuit and inflammatory pain (Figure 2D).

### 3.4 Optogenetic Activation of the PVT^Glut^→NAc Circuit Induces Nociceptive Behaviors in Naive Mice

**FIGURE 3:**
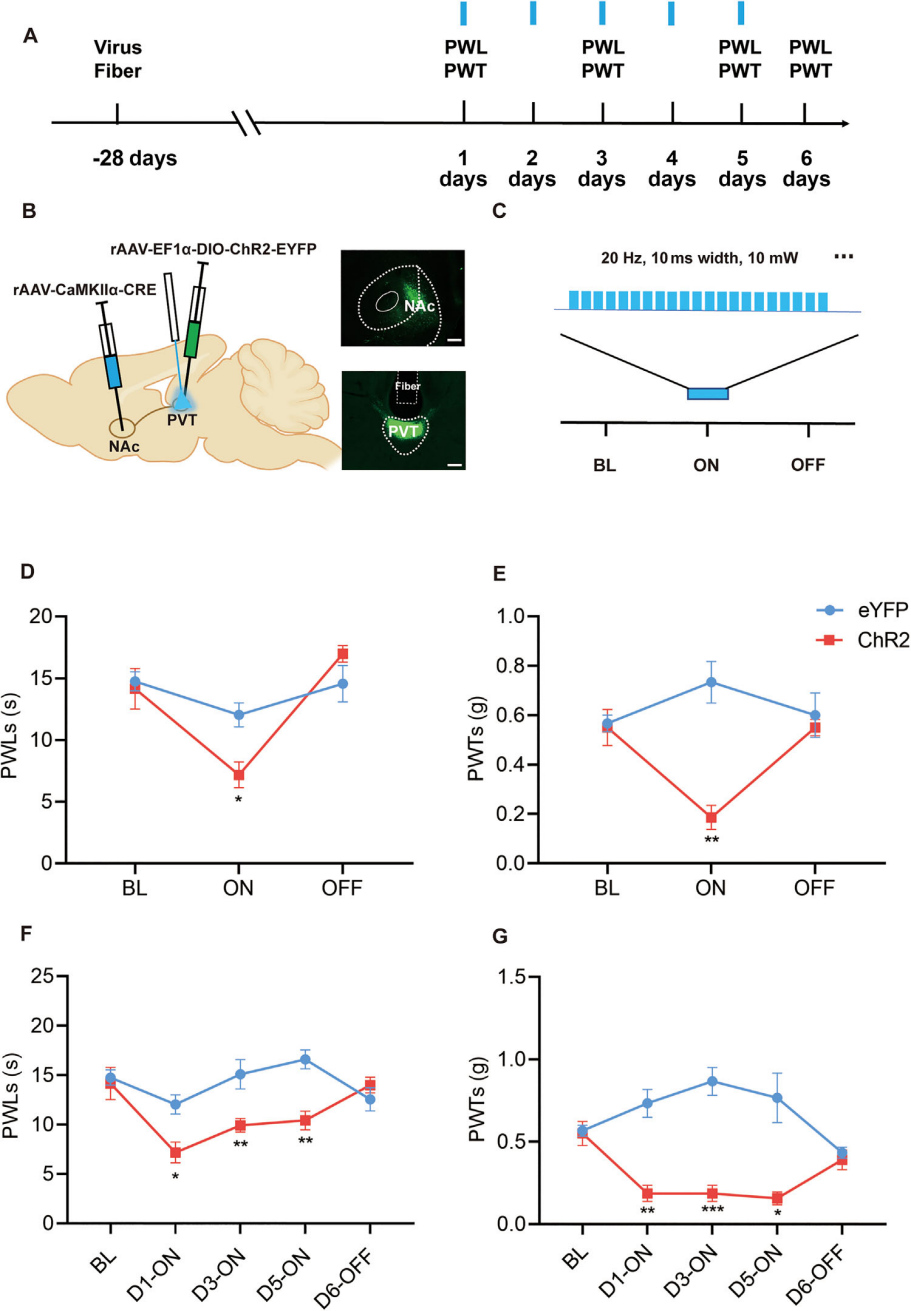
Activation of PVT^Glut^→NAc projection induces hyperalgesia‐like behaviors. (A) Experiment protocol. (B) Schematic illustration of virus injection and optical fiber implantation (Scale bar = 200 µm). (C) Optogenetic stimulation pattern. (D, E) PWLs and PWTs measured during BL (baseline)‐Laser ON‐laser OFF (mean ± SEM; two‐way ANOVA; *p < 0.05, **p < 0.01, *n* = 6, eight mice). (F and G) PWLs and PWTs measured during BL‐DAY1‐DAY3‐DAY5‐OFF (mean ± SEM; two‐way ANOVA; *p < 0.05, **p < 0.01, ***p < 0.001, *n* = 6, eight mice). These findings suggest that activation of the PVT^Glut^→NAc circuit can induce nociceptive behaviors in naive mice.

To further investigate whether optogenetic activation of the PVT^Glut^→NAc projection was sufficient to induce nociceptive behaviors in naive mice, we administered retrograde virus rAAV‐CaMKIIα‐Cre into the NAc, which allowed rAAV‐EF1α‐DIO‐ChR2‐EYFP (*n* = 8) for the channelrhodopsin‐2 (ChR2) group or rAAV‐EF1α‐DIO‐EYFP (*n* = 6) for the EYFP control group injected into the PVT to express, targeting PVT^Glut^→NAc projecting neurons and their axon terminals (Figure 3A,B). Optical fibers were implanted above the PVT for subsequent optogenetic stimulation. At 28 days post‐injection of the virus, the optogenetic stimulation was performed by employing an optic fiber connected to a laser light source that emitted a wavelength of 473 nm, facilitating ChR2 photo‐activation (Figure 3C). The experimental results show that through a single 1‐hour‐long stimulation, the activation of the PVT^Glut^→NAc circuit resulted in decreased PWLs and PWTs, with effects returning to the baseline level within 2 h after cessation of laser irradiation (Figure 3D,E). Then the PVT^Glut^→NAc circuit in mice was stimulated with continuous periodic blue laser light for 1 h per day over a period of 5 days. We did the behavioral assessment on first day, third day, and fifth day when the light stimulation time was full for an hour and similar results were observed in the somatic optogenetic experiment. Finally we did the behavioral assessment on the sixth day without light stimulation; painful behaviors were not found in the absence of light stimulation (Figure 3F,G). These findings suggest that activation of the PVT^Glut^→NAc circuit can induce nociceptive behaviors in naive mice.

### 3.5 The Inhibition of the PVT^Glut^→NAc Circuit Alleviates Nociceptive Behaviors in the Inflammatory Pain Mice Model

**FIGURE 4:**
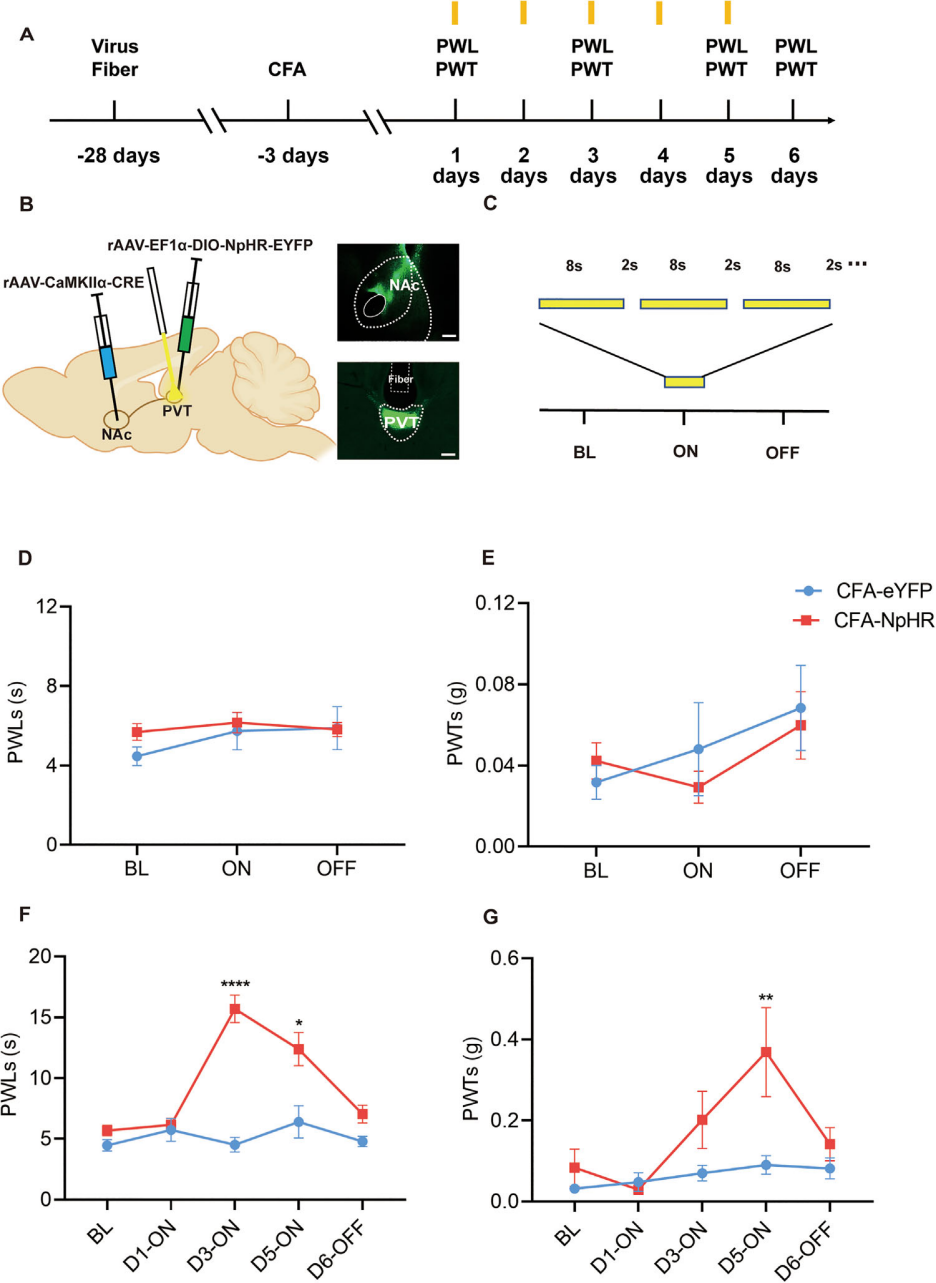
Inhibition of PVT^Glut^→NAc projection remits pain in inflammatory pain mice. (A) Experiment protocol. (B) Schematic illustration of virus injection and optical fiber implantation (scale bar = 200 µm). (C) Laser stimulation pattern. (D, E) PWLs and PWTs measured during BL‐Laser ON‐laser OFF (mean ± SEM; two‐way ANOVA; *n* = 6, eight mice). PWLs and PWTs in CFA mice were not ascended upon optogenetic inhibition of the PVT^Glut^→NAc neural circuit with single light stimulation. (F, G) PWLs and PWTs measured during BL‐DAY1‐DAY3‐DAY5‐OFF (mean ± SEM; two‐way ANOVA; *p < 0.05, **p < 0.01, ****p < 0.0001, *n* = 6, eight mice). These findings suggest that inhibiting the PVT^Glut^→NAc circuit with periodic light stimulation can alleviate pain in mice with inflammatory pain.

Next, we attempted to ascertain the analgesic effects of inhibiting the PVT^Glut^→NAc circuit in the mice with inflammatory pain. Similarly, we administered rAAV‐CaMKIIα‐Cre into the NAc and rAAV‐EF1α‐DIO‐NpHR3.0‐EYFP (*n* = 8) or rAAV‐EF1α‐DIO‐EYFP (*n* = 6) into the PVT to specifically express in PVT^Glut^→NAc projecting neurons and their axon terminals (Figure 4A,B). The optical fibers were implanted above the PVT for the subsequent optogenetic stimulation. After 25 days of virus injection, CFA was injected into the right hind paw of the mice. After an additional 3 days, optogenetic stimulation was performed using an optic fiber connected to a laser light source emitting 589 nm for NpHR photo‐activation (Figure 4C). Surprisingly, despite a single 2‐h stimulation, compared with the EYFP control group, no changes were observed in PWLs and PWTs upon optogenetic inhibition of the PVT^Glut^→NAc neural circuit (Figure 4D,E). The findings suggest that single light stimulation does not exert inhibitory effects on pain behavior in mice. Considering the activated state of neurons, it is plausible that brief light stimulation fails to reduce their excitability; therefore, we conducted periodic light stimulation. To examine the regulatory function of the PVT^Glut^→NAc circuit in mice on inflammatory pain, we applied continuous periodic yellow laser stimulation for 2 h daily over a span of 5 days. We conducted the behavioral assessment on the first, third, and fifth days and observed that optogenetic inhibition of the PVT^Glut^→NAc circuit significantly enhanced PWLs on Days 3 and 5, as well as PWTs on Day 5. Subsequently, we performed a behavioral assessment on the sixth day without light stimulation, revealing painful behaviors in the absence of light stimulation (Figure 4F,G). These findings suggest that inhibiting the PVT^Glut^→NAc circuit can alleviate pain in mice with inflammatory pain.

## 4 Discussion

Pain is a prevalent comorbidity in numerous clinical conditions, exerting an impact on both the physical and mental well‐being of patients while simultaneously imposing a significant burden on society (Cohen, Vase, and Hooten 2021). The mechanism underlying pain is rather intricate, involving the response of neural networks comprising multiple nuclei within the brain to noxious stimuli following complex information processing (Yang and Chang 2019). The rapid advancement of neuroscience has led to the emergence of increasingly sophisticated technologies, greatly facilitating the development of neural circuit research in areas such as pathological and physiological behavioral regulation, including pain.

Based on a substantial body of literature confirming the pivotal role of the PVT and NAc in pain modulation, our study aims to elucidate the crucial involvement of the PVT^Glut^→NAc circuit in the pain regulation. The PVT consists predominantly of glutamatergic neurons; therefore, the neurons affected by viral vectors carrying the CaMKIIα promoter can be considered as putative glutamatergic neurons in this study. Our findings suggest that in the presence of inflammatory pain, both PWLs and PWTs are reduced in the right hind limb of mice compared to the control group. The expression of c‐Fos protein was elevated in the PVT of mice with CFA‐induced inflammatory pain, providing evidence for heightened neuronal activity within the during states of inflammatory pain. The presence of glutamatergic neurons projecting to NAc was observed in the PVT following retrograde injection of a fluorescently labeled virus into the NAc. The glutamatergic neuronal projection relationship between the PVT and the NAc was once again confirmed at the anatomical level, aligning with previous findings in the literature. The activation of the PVT^Glut^→NAc glutamatergic circuit through optogenetic techniques can elicit changes in PWLs and PWTs that resemble those observed in states of inflammatory pain. The inhibition of the PVT^Glut^→NAc circuit in successive cycles can lead to the restoration of PWLs and PWTs in mice with CFA‐induced inflammatory pain to levels comparable to those seen in normal levels. The aforementioned findings suggest that the PVT^Glut^→NAc circuit plays a pivotal role in the regulation of inflammatory pain. The collective findings from these studies, characterized by high specificity, have firmly established a robust causal relationship between the hyperactivity of the PVT^Glut^→NAc pathway and pain sensation. It is worth mentioning that G. C. Zhang et al. (2023) found that PVT^Glut^→NAc neuronal activity increased in response to acute thermal/mechanical stimuli and persistent inflammatory pain, which is consistent with our conclusions.

The NAc is a crucial neuroanatomical region that governs animal behavior and serves as the hub for signal integration from various brain regions, including the VTA, hippocampus, PFC, thalamus, and other interconnected areas (Russo and Nestler 2013). Although the distribution of inputs received by different cell types in the NAc exhibited a high degree of similarity, notable variations were observed in the input patterns across distinct brain regions within different subregions of the NAc. Specifically, cortical structures such as the orbital cortex, insular cortex, ventral cortex, and PFC displayed a preference for projecting to the NAc core. Conversely, subcortical structures like the hippocampus and lateral hypothalamus exhibited a greater propensity to send fibers projecting towards the NAc shell (Z. Li et al. 2018). The distinct patterns of differential projection between various subregions of the NAc and other brain regions may underlie the functional specialization observed across the different NAc territories (Floresco et al. 2006).

The NAc is a crucial brain region involved in the regulation of animal behavior. The PFC and its projected NAc have been found to play a crucial role in pain development, as evidenced by numerous studies. Furthermore, chronic pain can disrupt the functional connectivity between the PFC and other brain regions (Lee et al. 2015). The activation of glutaminergic receptors in neurons located in the PFC leads to a reduction in pain sensation (Millecamps et al. 2007). It has also been confirmed that the medial PFC (mPFC)→NAc neural circuit has a regulatory effect on inflammatory pain (Goffer et al. 2013; Navratilova and Porreca 2014). In addition, the VTA is also a crucial brain region involved in pain and depression, with its dopaminergic neurons and projections to various mesolimbic reward circuit regions playing a critical role in both pain perception and related emotional experiences. The levels of dopamine released into the NAc are altered in chronic pain, indicating potential involvement of the reward circuitry formed by midbrain dopaminergic neurons and the NAc in pain modulation (Bannister et al. 2017; Kato et al. 2016; Porreca and Navratilova 2017). The glutaminergic pathway from the amygdala to the NAc, in conjunction with dopamine signaling in the NAc, facilitates motivational behavioral responses (Stuber et al. 2011).

The PVT plays a pivotal role in the processing of sensory information in the brain (Cheng et al. 2017; Y.‐T. Chang et al. 2019; Chen et al. 2010). The functional role of thalamic circuits, consisting of the PVT glutamatergic neurons and their associated brain regions, in the mediation of pain processing has been elucidated by recent studies. The PVT is a thalamic midline nucleus comprised of glutamatergic neurons, serving as the central hub for detecting and responding to both external physical and mental stimuli (Millan, Ong, and Mcnally 2017; Ehling et al. 2018). The injection of NBQX, a glutaminergic receptor antagonist, into the NAc, can effectively inhibit aversive behavior induced by photogenetic stimulation of the PVT→NAc circuit. This finding confirms the anatomical correlation between the PVT^Glut^→NAc circuit and its significant role in animal behavior. In addition, it reveals the involvement of the PVT^Glut^→NAc circuit in mediating opioid dependence (Zhu et al. 2016). Due to the fact that opioids can cause adverse reactions such as nausea, vomiting, respiratory depression, and itching, and can also lead to addiction, other types of drug development for analgesia are extremely necessary (Stein 2018). Glutamate receptor antagonists are a good direction for research. In conclusion, it could be identified as a promising target within the brain for future pain treatment.

In the present study, we exclusively used male mice to investigate the role of a neural circuit from PVT to NAc in inflammatory pain. Historically, most preclinical pain research was conducted exclusively in male animals. However, recent studies that included females have revealed significant sex differences in the physiological mechanisms underlying pain, including sex‐specific involvement of different genes and proteins as well as distinct interactions between hormones and the immune system that influence the transmission of pain signals. The focus of the IASP 2024 Global Year is on sex and gender disparities in pain. To better understand why this happens and consider the implications for how we manage pain, researchers need to investigate the underlying causes of sex/gender‐related inequalities and inequities in pain occurrence.

## 5 Conclusion

The present study indicates that the PVT→NAc projection circuit plays a role in regulating inflammatory pain, with the specific involvement of glutamatergic neurons in the PVT projecting to the NAc as the cellular mechanism mediating the regulatory function.

## Author Contributions

**Xi Liu**: behavioral and biochemical experiments, data curation, formal analysis, investigation, writing–original draft, writing—review and editing. **Xi Zhang**: biochemical experiments, data curation, formal analysis, writing—review and editing. **Dongxu Wang**: biochemical experiments, data curation, formal analysis, writing—review and editing. **Ya Cao**: investigation, software, writing—review and editing. **Ling Zhang**: investigation, methodology. **Zhonghua Li**: investigation, methodology. **Qin Zhang**: investigation, formal analysis. **Yu Shen**: investigation, methodology. **Xian Lu**: investigation. **Keyu Fan**: investigation. **Mingxia Liu**: investigation. **Jingqiu Wei**: writing—review and editing, supervision. **Siping Hu**: funding acquisition, writing—review and editing, supervision. **He Liu**: funding acquisition, writing—review and editing, supervision.

## Conflicts of Interest

The authors declare no conflicts of interest.

### Peer Review

The peer review history for this article is available at https://publons.com/publon/10.1002/brb3.70218.

## Data Availability

The data that support the findings of this study are available from the corresponding author upon reasonable request.
